# Supplementary material for: Chip-scale high-peak-power semiconductor/solid-state vertically integrated laser
Source: Nat Commun. 2022 Oct 1;13:5774. doi: 10.1038/s41467-022-33528-x (PMC9526722; doi:10.1038/s41467-022-33528-x)
Supplement: Supplementary file 2 — Lasing Reporting Summary [file 41467_2022_33528_MOESM2_ESM.pdf]

nature research

Corresponding author(s): Masanao Kamata

Lasing Reporting Summary

Nature Research wishes to improve the reproducibility of the work that we publish. This form is intended for publication with all accepted papers reporting claims of lasing and provides structure for consistency and transparency in reporting. Some list items might not apply to an individual manuscript, but all fields must be completed for clarity.

For further information on Nature Research policies, including our data availability policy, see Authors & Referees.

Experimental design

Please check: are the following details reported in the manuscript?

1. Threshold

Plots of device output power versus pump power over a wide range of values indicating a clear threshold

☒ Yes
☐ No

See Fig. 4a and Fig. 5b.

2. Linewidth narrowing

Plots of spectral power density for the emission at pump powers below, around, and above the lasing threshold, indicating a clear linewidth narrowing at threshold

☒ Yes
☐ No

See Fig. 4d and Fig. 5e. Only spectrum above lasing threshold is measured due to characteristics of passively Q-switched laser oscillation.

Resolution of the spectrometer used to make spectral measurements

☒ Yes
☐ No

See Results: Passively Q-switched Laser Demonstration.

3. Coherent emission

Measurements of the coherence and/or polarization of the emission

☒ Yes
☐ No

As indications of coherent emission, see Fig. 5e and Fig. 5f. Polarization control is a future work for us and it is not reported in this paper.

4. Beam spatial profile

Image and/or measurement of the spatial shape and profile of the emission, showing a well-defined beam above threshold

☒ Yes
☐ No

See Fig. 5f.

5. Operating conditions

Description of the laser and pumping conditions
Continuous-wave, pulsed, temperature of operation

☒ Yes
☐ No

See Results: Passively Q-switched Laser Demonstration.

Threshold values provided as density values (e.g. W cm^{-2} or J cm^{-2}) taking into account the area of the device

☐ Yes
☒ No

Threshold current density (A/cm2) values can be easily calculated from the threshold current and the oxide layer aperture, which can be found in Results: Passively Q-switched Laser Demonstration and Methods: Device Structure.

6. Alternative explanations

Reasoning as to why alternative explanations have been ruled out as responsible for the emission characteristics
e.g. amplified spontaneous, directional scattering; modification of fluorescence spectrum by the cavity

☐ Yes
☒ No

There is no other explanation for the emission characteristics other than passively Q-switching.

7. Theoretical analysis

Theoretical analysis that ensures that the experimental values measured are realistic and reasonable
e.g. laser threshold, linewidth, cavity gain-loss, efficiency

☒ Yes
☐ No

See Results: Passively Q-switched Laser Simulation.

8. Statistics

Number of devices fabricated and tested

☒ Yes
☐ No

We fabricated and tested four chip-scale devices, and it was confirmed that they showed similar characteristics.

Statistical analysis of the device performance and lifetime (time to failure)

☒ Yes
☐ No

See Fig. 5c and Fig. 5g and related explanation in Results: Passively Q-switched Laser Demonstration.
